# Physiotherapists’ perceptions of and willingness to use telerehabilitation in Kuwait during the COVID-19 pandemic

**DOI:** 10.1186/s12911-021-01478-x

**Published:** 2021-04-08

**Authors:** Sarah Ibraheem Albahrouh, Ali Jasem Buabbas

**Affiliations:** 1 Department of Physical Therapy, Faculty of Allied Health Sciences, Kuwait University, Kuwait City, Kuwait; 2 Department of Community Medicine and Behavioural Sciences, Faculty of Medicine, Kuwait University, Kuwait City, Kuwait

**Keywords:** Physiotherapists, Telerehabilitation, Perceptions, COVID-19 pandemic

## Abstract

**Background:**

Telerehabilitation has been considered a suitable alternative healthcare delivery system during the COVID-19 outbreak, and many studies have promoted its feasibility in delivering physical care to patients who live with pain and disability. Physiotherapists’ perceptions and willingness are two key factors that influence the provision of remote physiotherapy.

**Aim:**

To investigate physiotherapists’ perceptions of and willingness to use telerehabilitation in Kuwait during the COVID-19 pandemic and to explore the barriers that may hinder the use of telerehabilitation in this sector.

**Methods:**

The following methods were used: (1) a cross-sectional survey and (2) face-to-face semi-structured interviews. In the cross-sectional survey, an electronic questionnaire was sent to 747 physiotherapists who were working in the governmental health sector. The questionnaire included four sections: perceptions of telerehabilitation, comfort with technology, willingness to use telerehabilitation, and barriers to using telerehabilitation. Six interviews were conducted with physiotherapy managers to explore the barriers and facilitators of telerehabilitation practice.

**Data analysis:**

In this study, descriptive data analysis was conducted, and a cross-tabulation technique was used to find the associations between the variables, in which chi-square tests were used to identify the significance of the results, where *p* < 0.05. Thematic content analysis was used for the interviews.

**Results:**

Only 273 completed questionnaires were received, giving a response rate of 36.5%. Most of the respondents (237; 86.8%) considered telerehabilitation a viable option to deliver healthcare to patients during the COVID-19 pandemic. In spite of the lack of information and communication technology (ICT) (156; 57.1%), 89% (243) of the respondents were willing to integrate telerehabilitation into their conventional practice, as cultural and social norms were not against the use of telerehabilitation systems. The results indicate that the more the physiotherapists used the internet and email in their work and the more comfortable they were with technology, the more willing they were to use telerehabilitation systems (*p* = 0.01). The physiotherapy managers reported that patients’ privacy and the confidentiality of their data were considered barriers.

**Conclusion:**

In Kuwait, physiotherapists show overall positive perceptions towards and a willingness to use telerehabilitation to facilitate patients’ access to physiotherapy services. There are several barriers to employing telerehabilitation. Accordingly, recommendations are suggested.

**Supplementary Information:**

The online version contains supplementary material available at 10.1186/s12911-021-01478-x.

## Introduction

The outbreak of coronavirus disease (COVID-19) has changed many aspects of people’s lives and has pushed governments and health authorities to implement several protective measures, such as social distancing, to minimize the risk of exposure. The Ministry of Health of Kuwait has minimized access to outpatient rehabilitation clinics and has postponed the treatment of non-urgent conditions. Consequently, almost all physiotherapy services have been suspended. The impact of the COVID-19 pandemic has forced healthcare decision-makers to consider the risks associated with reducing the healthcare and rehabilitation services for non-COVID patients, as well as to consider alternative modes for delivering healthcare, such as telehealth [1]. Telehealth refers to the use of telecommunications and virtual technology to deliver healthcare, including patient rehabilitation. Currently, in this difficult time, there is a need for an adequate approach to deliver healthcare and to ensure access to clinical services [2]. The global pandemic has required the physiotherapy profession to consider digital physiotherapy practice and telehealth as a method to deliver healthcare services [3, 4]. The World Confederation for Physical Therapy (WCPT) has promoted the use of telerehabilitation in the profession to improve accessibility [5], with a number of national organizations providing resources and advice on implementing such services during the COVID-19 pandemic [6–8].

Several studies have highlighted the perspectives and acceptance of clinicians and healthcare providers in regard to telerehabilitation use [9–12]. Overall, positive impressions and high rates of satisfaction among clinicians and healthcare providers were reported in these studies. This high level of satisfaction was owing to the advantages of telerehabilitation, which include: (1) increasing the flexibility of work hours and locations; (2) reducing worries about the availability of space in many clinical settings; (3) providing a cost-effective way to deliver care; and (4) reducing patients’ travel time to attend clinics [10, 11, 13]. A previous study also showed that the confidence of physiotherapists (PTs) in providing assessment and treatment via telerehabilitation influences their use of the technology; moreover, as an example, patient–clinician rapport in a spinal care clinic significantly improved as the clinicians gained experience in telerehabilitation [11].

PT–patient physical contact is considered a challenge to telerehabilitation use [14]. Even though there are highly sophisticated telerehabilitation systems, such as the Australian eHABTM system, which combines real-time videoconferencing and accurate remote diagnosis compatible with conventional face-to-face intervention outcomes, an adequate alternative for hands-on skills has not been found [15, 16]. Inadequate infrastructure (such as poor internet coverage, inadequate internet services, or network failure), the unavailability of telecommunication devices, and a lack of computer literacy have also been identified as barriers to the implementation of telerehabilitation [9, 10, 17]. Worries about compromising patient safety and privacy when using telerehabilitation are further barriers to teleservices [10, 11, 13]. Legal and regulatory restrictions also have an impact on providing online consultations, preventing many PTs from using telerehabilitation [13, 18].

To address the identified challenges, previous studies have recommended that governments should design pragmatic policies to promote telerehabilitation services [14, 19, 20]. It is imperative to understand the perspectives of healthcare providers regarding the implementation of telerehabilitation to ensure the long-term use of such systems [21].

According to the extant literature and the publications of the previously mentioned professional bodies, remote healthcare may help to ensure safe practices; however, there is a knowledge gap in the literature concerning PTs’ perceptions of and willingness to use telerehabilitation during the COVID-19 pandemic. Therefore, the aim of this study was to investigate PTs’ perceptions of and willingness to use telerehabilitation in Kuwait during the COVID-19 pandemic, in addition to exploring the barriers to the successful implementation of telerehabilitation in the physiotherapy sector.

Our hypothesis was that PTs are willing to use telerehabilitation and have positive perceptions in relation to delivering physiotherapy services at a distance during the COVID-19 pandemic. This study provides preliminary information regarding PTs’ current perceptions and willingness regarding the use of telerehabilitation, which will aid in decision-making around PTs offering telerehabilitation services on a routine basis during and after the COVID-19 pandemic.

## Methods

The design of this study was cross-sectional. Both quantitative and qualitative methods were employed in order to achieve the aim of the study, which was conducted over a period of just under 3 months (1 June to 27 August 2020).

### Ethical considerations

The study was approved by the Ministry of Health’s Standing Committee for the Coordination of Health and Medical Research in the State of Kuwait (1478/2020). Written consent was obtained from each participant in this study, and the participants were informed that their participation was voluntary and that they could withdraw at any time.

### Participants

For the quantitative approach, convenience sampling was conducted. A web survey was developed via Google Docs to collect data from PTs. The director of physical therapy administration at the Ministry of Health took responsibility for distributing the link to the questionnaire, in order to increase the response rate. This was done via a WhatsApp (a smartphone messaging application) group that included all of the PTs working in the governmental sector in Kuwait. Furthermore, a soft reminder message was sent once a week; the data collection process took about a month. This message informed the professional group of the importance of the study’s outcomes in the physiotherapy arena. In the questionnaire, a confidentiality statement was shown after the title and aim of the study, informing the respondent that their identity would be kept anonymous.

In total, 273 out of 747 eligible PTs completed the questionnaire and took part in this study. All participants had at least 1 year of clinical experience and were currently working in governmental sector hospitals across the six regions of Kuwait, namely: Capital, Ahmadi, Farwaniyah, Sabah, Hawally, and Jahra. In addition, participants who could not read and understand English were excluded from the study.

For the qualitative approach, the data collection was managed and performed by the study’s main investigator (a PT/academic), who approached the director of the physical therapy administration to facilitate the process of inviting all the managers of the physiotherapy departments within the governmental sector to voluntarily participate in the interviews. Accordingly, semi-structured interviews were pre-arranged with six managers, and these were then conducted by the study’s main investigator, who has significant experience in this type of approach.

The interviews involved managers who had at least 20 years of work experience and at least 5 years of administrative experience. The interviews with the physiotherapy managers were intended to provide a good understanding of the current situation of telerehabilitation practice, including the difficulties faced and ways to overcome them.

### Instruments

#### Questionnaire

A self-reported questionnaire was used in this study, which had been previously used [22], and included 25 items divided into five sections (see Additional file 1). Section 1 focused on demographic data, including age, gender, nationality, years of experience, and area of work. Section 2 aimed to explore the technological background of the PTs and their skills related to using information and communication technology (ICT) and telerehabilitation methods in their workplaces. Sections 3, 4, and 5 were designed to assess the PTs’ perceptions of telerehabilitation, comfort with technology, willingness to use telerehabilitation, and perceptions of the barriers that could be faced while using telerehabilitation. The answers in these three sections were provided using a four-point Likert scale (from strongly disagree to strongly agree). Some of the questionnaire items were adapted to suit the physiotherapy profession.

The questionnaire was piloted with ten PT specialists to test the suitability and readability of the items. Minor feedback was provided, and modifications were made accordingly, which included adding the items “years of experience” and “area of work”.

#### Interviews

Semi-structured, in-depth, face-to-face interviews were conducted with physiotherapy managers, which required pre-arranged meetings. An interview topic guide was developed based on the aim of the study, utilizing questions from similar previous research [14]—see Table 1. All participants were informed about the aim of the interviews and the fact that the interviews would be audio-recorded. At the start of each interview, the participant’s age, gender, education level, years of work and administrative experience, and department specialty were obtained. Afterwards, open-ended follow-up questions were used to obtain detailed descriptions. The researcher used probes to guide the conversation in an appropriate way. The audio-recorded interviews were transcribed verbatim.

**Table 1:** Interview questions

1. Do you use telerehabilitation in your department? If the answer is Yes, proceed to question 2. If the answer is No, proceed to question 4
2. What are the reasons for using telerehabilitation in your department?
3. How have PTs used the telerehabilitation system in your department during the pandemic?
4. What are the barriers to implementing telerehabilitation in your department?
5. What are the factors facilitating the use of telerehabilitation in your department?
6. What is required to implement successful telerehabilitation practice in your department?

### Data analysis

Descriptive data analysis was conducted, which included calculating the frequencies and percentages of the participants’ demographic data [23]. A cross-tabulation technique was used to find the associations between the variables, in which chi-square tests were used to identify the significance of the results, where *p* < 0.05 [23].

**Fig. 1:**
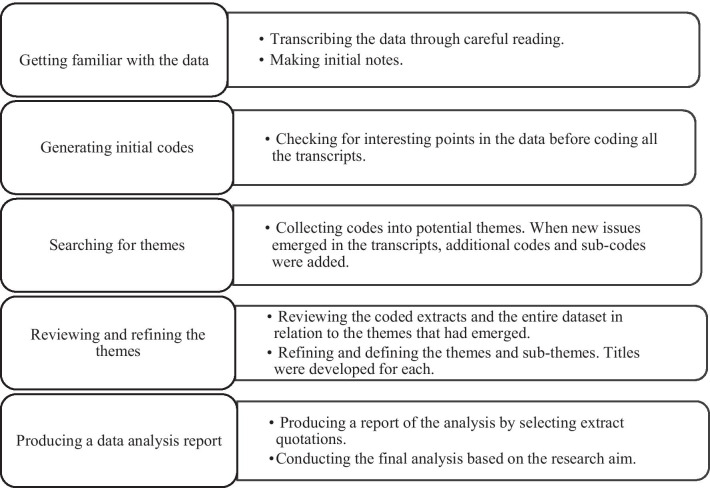
Process of the thematic content analysis

The interviews, original transcriptions, and data analysis were in English. Thematic content analysis was used by the main investigator to analyse the transcripts (see Fig. 1) [24]. The coding process was done manually using word-processing software. Rigor was ensured through participant feedback [25], whereby a couple of randomly chosen transcripts with codes and quotations were sent back to the participants to check the accuracy of the contents. The reliability of the data was also checked through peer-checking [26], whereby the codes and subcodes of the primary researcher and another researcher, who were both familiar with qualitative studies, were compared for the first two transcripts. There were slight differences, which were discussed and resolved during discussion meetings.

## Results

### Questionnaire results

#### Participant profile

The demographic data of the participants is shown in Table 2. Only 273 completed questionnaires were received, giving a response rate of 36.5%. The results show that approximately half of the respondents were non-Kuwaiti (55.7%), and over half of the respondents were female (65.6%). Most of the respondents were aged 35–50 years old (59%). The results also show that most of the respondents were PT specialists and senior physiotherapy specialists (43.2%).

**Table 2:** Participant demographics

Demographic variable	No. (%)
Gender	
Male	94 (34.4)
Female	179 (65.6)
Age	
Less than 35	94 (34.4)
35–50	161 (59)
51–60	9 (3.3)
Above 60	9 (3.3)
Nationality	
Kuwaiti	121 (44.3)
Non-Kuwaiti	152 (55.7)
Hospital setting	
General hospital	104 (38.1)
Rehabilitation hospital	110 (40.3)
Specialist hospital	59 (21.6)
Professional rank	
Physiotherapy/senior physiotherapy practitioner	100 (36.6)
Physiotherapy/senior physiotherapy specialist	118 (43.2)
Superintendent PT	55 (20.1)

#### Background information

Some of the respondents (30.8%) reported that they always used a computer at work, 24.5% stated that they sometimes used the internet at work, and 23.1% said that they sometimes used email at work. Half of the respondents (51.6%) reported that they never used any telerehabilitation methods in their workplaces, while only 2.6% of the respondents said that they always used telerehabilitation methods.

#### Perceptions of telerehabilitation

**Fig. 2:**
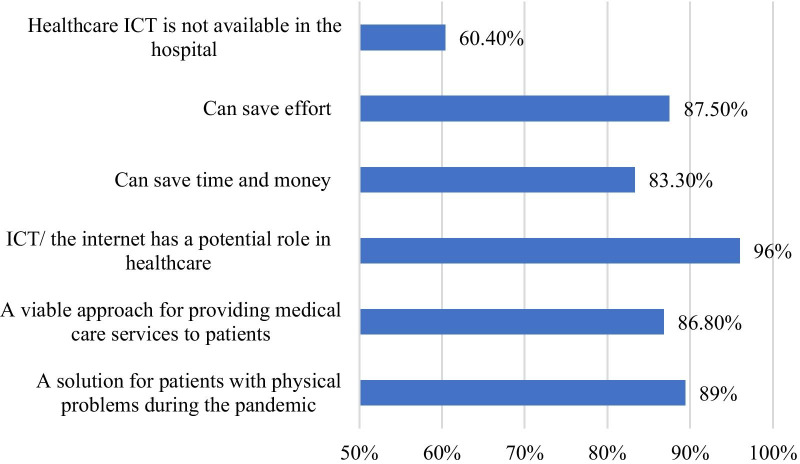
PTs’ perceptions of telerehabilitation systems

Figure 2 shows the results of the PTs’ perceptions of telerehabilitation systems. The results demonstrate that most of the respondents agreed that telerehabilitation systems could be a solution for handling patients with physical problems during the COVID-19 pandemic. However, more than half of the respondents stated that the application of healthcare ICT is not available in their hospitals.

#### Comfort with technology

Most of the respondents agreed that they could generally trust technology to work (87.9%), and they were happy using ICT/the internet for the purposes of patient care and education (91.6%). Most of the respondents (93.4%) declared that they were generally comfortable using ICT/the internet for storing, retrieving, and communicating patient information with other healthcare institutions. Most of the respondents (81%) stated that cultural and social norms did not prohibit the use of telerehabilitation systems.

#### Willingness to use telerehabilitation

**Fig. 3:**
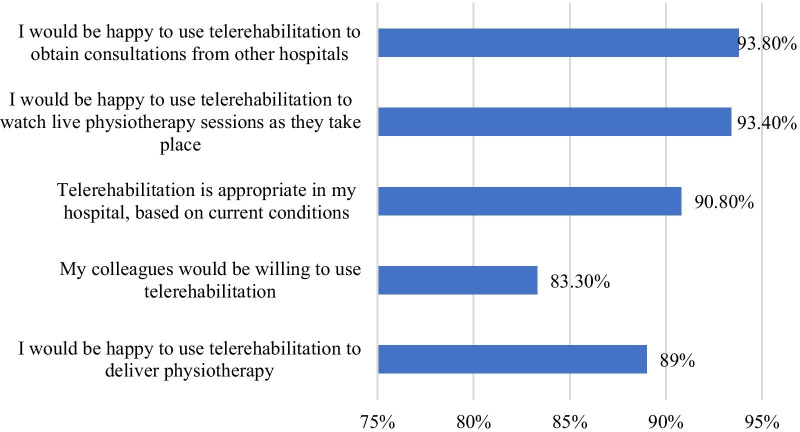
Willingness of the respondents to use telerehabilitation systems

Figure 3 shows the willingness of the respondents to use telerehabilitation systems. The results illustrate that the majority of the respondents (93.8%) were happy to use telerehabilitation systems to obtain consultations from other medical centres/hospitals. Furthermore, most of the respondents (89%) were willing to deliver physiotherapy via telerehabilitation.

A significant association (0.021) was identified between age and willingness to use telerehabilitation systems. PTs aged 35–50 years old were more willing to use telerehabilitation than those who were less than 35 years old and those who were more than 50 years old. In addition, there was a significant association (0.016) between professional rank and willingness to use telerehabilitation systems. Specialist and senior PTs were most willing to use telerehabilitation systems. No associations were found between willingness to use telerehabilitation and other demographic data, such as gender and nationality. A significant association was also found (0.01) between use of technology and willingness to use telerehabilitation, which indicated that the more the PTs used the internet and email in their work and the more comfortable they were with technology, the more willing they were to use telerehabilitation systems.

#### Barriers to the use of telerehabilitation systems

**Fig. 4:**
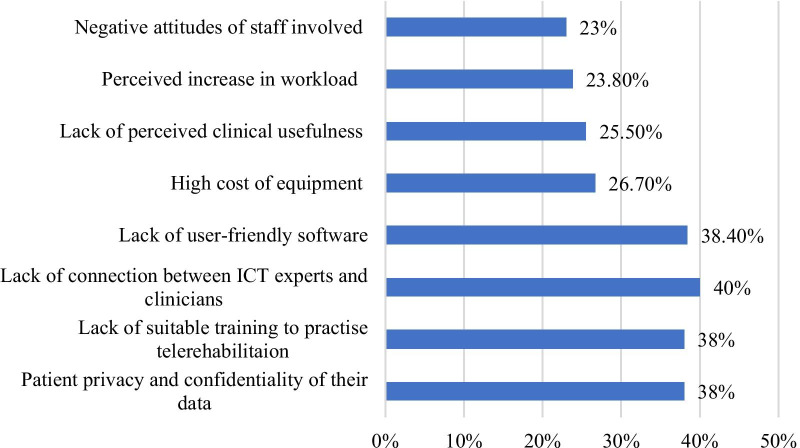
Barriers to the use of telerehabilitation systems

Figure 4 shows the barriers identified by the participants as obstacles to telerehabilitation implementation in their hospitals. The results illustrate that the most common barrier identified by the respondents was the lack of connection between ICT experts and clinicians, which could lead to the inappropriate selection of software that is not user-friendly.

### Interview results

#### Demographic data

Six managers of physiotherapy departments, five female and one male, agreed to take part in interviews regarding their perceptions of telerehabilitation practice in Kuwait during the COVID-19 pandemic. Two of the managers were from general hospitals, three were from specialized centres, and one was from the main administration. The age range was 47–66 years old. Five of the managers had bachelor’s degrees, while one had a master’s degree. The work experience of the participants ranged from 20 to 35 years, and their administrative experience ranged from 5 to 30 years.

#### Interview themes

Five themes emerged from the data: telerehabilitation practices, the need for telerehabilitation systems, factors facilitating telerehabilitation in clinical practice, barriers to successful telerehabilitation use, and telerehabilitation implementation requirements.

#### Telerehabilitation practices

All the managers said that they used telerehabilitation systems in their departments, and they confirmed the usefulness of telerehabilitation techniques during the pandemic, particularly when the Ministry of Health locked down the outpatient departments. The PTs in their departments made appointments for virtual sessions via their personal smartphones, iPads, and computers. Only one manager said that their hospital IT department had provided a landline with a smartphone and the internet. The PTs used different platforms, such as WhatsApp, Zoom, and Skype, to conduct video-call sessions. Most of them used WhatsApp because it is handy and widely available for both PTs and patients. Only 50% of the managers said that their PTs initially contacted patients to assess their suitability for the virtual sessions. If the patient needed direct care and hands-on physiotherapy, then they would be scheduled an initial in-person appointment. After that, virtual sessions were recommended for follow-up.

“By sending messages through WhatsApp when we get the referral… we send them messages like a questionnaire about their movement and activities, if they use any kind of assistive device. After that initial assessment, the therapist will decide whether to continue online or to bring the patients into the department to practise some hands-on interventions and teach the patients and their caregivers the exercises.” (M1)

All the managers said that they did not follow any protocols or guidelines and did not have any rules or regulations regarding telerehabilitation practice.

#### The need for telerehabilitation systems

All the managers stated that because of the pandemic, their departments were offering telerehabilitation to prevent the spread of COVID-19 and to ensure the safety of both patients and staff, as well as to provide safe healthcare services for vulnerable patients, such as elderly people.

“Because we don’t want to spread the disease… some of these were old patients… so we started using this technology to help the patients to have their treatment at home.” (M4)

The managers mentioned that telerehabilitation systems can be used to reduce waiting lists, manage many chronic non-urgent cases from a distance, provide flexible appointment times, and save patients the effort of attending clinics. Moreover, telerehabilitation systems can encourage patients to perform exercises at home. The results show that all the managers indicated the usefulness of telerehabilitation during the COVID-19 pandemic and confirmed the willingness of their staff to use it after the pandemic.

“We have a long waiting list in the outpatient department… because patients are continually coming in with chronic cases… these kinds of patients, you might follow up with them through telerehabilitation… you might give them the instructions… they don’t have to come to the department… in this situation, let’s give the chance to acute and new cases… and other patients come from a distance… some patients have missed sessions because of traffic, or they don’t have transportation…” (M6)

#### Factors facilitating telerehabilitation in clinical practice

All the managers indicated that most of the PTs in their departments were willing and happy to use telerehabilitation through video-calls. They said that most of the PTs could use the technology and were happy and satisfied with the patient outcomes. The managers also indicated that the PTs had become more motivated towards and interested in telerehabilitation over time.

“Honestly… in the beginning, I didn’t expect this cooperation and acceptance from the staff in using telerehabilitation, especially as it is a new kind of practice… but over time and with experience… the PTs were happy and accepting of the idea of this kind of practice… especially when they saw the results and outcomes with their patients… they became more interested in this practice.” (M3)

All the managers stated that patients were happy and satisfied with the telerehabilitation services offered, especially well-educated patients and caregivers, those who were more cooperative with the PTs during the video-calls, and those who were more familiar with technology. Most of the patients committed to attending the virtual sessions, following the instructions, and practising the exercises at home.

“We have had feedback from some departments that patients have been very cooperative regarding telerehabilitation in terms of not only attending the virtual sessions but also implementing the treatment from a distance.” (M6)

Only 50% of the managers stated that their hospital management teams were happy with the telerehabilitation system. One manager stated that the hospital management team was looking at highlighting the successful outcomes of the telerehabilitation service, in order to encourage other departments in the hospital to use the same remote healthcare technique.

#### Barriers to successful telerehabilitation use

All the managers indicated that the main barriers hindering the successful use of telerehabilitation were the unavailability of adequate equipment, poor network coverage, and a lack of hospital and IT support.

“The network coverage, we provide ourselves… we don’t have a network in our hospital… as we don’t have an IT department in our hospital, unfortunately”. (M3)

Moreover, the managers said that telerehabilitation has many disadvantages, such as difficulty in detecting some physical problems, a lack of patient and therapist privacy, a lack of hands-on interventions, and a lack of perceived clinical effectiveness. The managers stated that some of their PTs had not accepted the use of telerehabilitation because they did not trust the effectiveness of telerehabilitation in identifying some patient problems, so they preferred in-person sessions.

“Over time, using a technology becomes easier… but physiotherapy practice itself difficult… as I said, some of our PTs want to put their hands on patients… and we must assess patients to know their problems… a video-call is not enough… PTs and other medical staff may not trust telerehabilitation and its effectiveness… The PTs themselves say that being hands on is essential and beneficial.” (M5)

Two of the managers stated that there were cultural and social barriers. Gender issues might have an impact on video-call sessions if female PTs refuse to conduct virtual sessions in the presence of male caregivers. Moreover, Kuwaiti society may not accept the idea of telerehabilitation because Kuwaitis may prefer physical modalities and hands-on interventions. Alternatively, they may not trust technology or may lack knowledge about the role of technology in rehabilitation.

“The only thing that is obvious is the refusal of female PTs to appear in video-calls because they might think that patients or their relatives will take their pictures or record videos of them, and, you know, most of them are married and their families refuse for anyone to see their faces. Therefore, the female PTs won’t show their faces in video-calls, and they will only instruct. If the male PTs appear in video-calls, they demonstrate the exercises for the patients.” (M1)

The results reveal that older and poorly educated patients were also considered barriers because they have difficulties in using smart technology and understanding the techniques that could be used through specific applications. Also, they might not react correctly to the instructions of PTs delivered through telerehabilitation systems. One of the managers mentioned a solution to overcome this barrier, which is to ask the patient to request one of their technology-savvy relatives to provide support during the virtual session.

#### Telerehabilitation implementation requirements

All the managers indicated that they needed full support from the hospital management and the Ministry of Health to facilitate the implementation of telerehabilitation in their physiotherapy departments. They needed facilities such as ICT support, smartphones, iPads, advanced computers with cameras, and adequate network connections.

“IT and higher management support… that’s why I need the outcomes from the telerehabilitation sessions so I can raise a report to show the senior management that it’s useful and beneficial. Not only to the senior management but also to the doctors and the PTs themselves.” (M6)

The managers also mentioned that guidelines, policies, and protocols are required to protect both patients and staff from legal allegations and to ensure safe and effective telerehabilitation practice.

“I think there should be some kinds of written instructions or official guidelines and protocols about telerehabilitation practices… like which kinds of patients need telerehabilitation, how to use it for different cases, how to organize and manage patients, which kinds of platforms we should use, and the rules and regulations so that all PTs can follow these guidelines.” (M2)

All the managers emphasized the importance of awareness programmes regarding telerehabilitation for healthcare workers, hospital administrators, and the public to increase the trust in and acceptance of telerehabilitation.

“Trust and awareness… we need an awareness and education programme for telerehabilitation… yes, they need to trust that telerehabilitation will give the same benefits as conventional practice… if they see the benefits, they may accept it… it is a new thing… everything is new at the beginning and may not be accepted… therefore, we need orientation programmes on the role of PTs in telerehabilitation.” (M6)

The managers also indicated the importance of training programmes, workshops, and trial systems to upgrade the level of telerehabilitation practice among PTs during and after the COVID-19 pandemic.

## Discussion

This study found that the PTs surveyed had positive perceptions of telerehabilitation and showed an interest in learning or improving the skills necessary to implement telerehabilitation in their practice, despite the lack of adequate infrastructure and support to implement telerehabilitation.

### PTs’ perceptions of telerehabilitation use

Most of the PTs agreed that telerehabilitation offers a practical solution to provide physiotherapy services to patients, particularly during the COVID-19 pandemic. However, telerehabilitation requires ICT facilities that are not currently available in most of the hospitals. This finding is consistent with previous studies wherein PTs reported that limited access to technical support at their facilities was behind them not adopting telerehabilitation [10, 14]. In contrast, PTs in Saudi Arabia reported lower utilization of telerehabilitation services by patients during the COVID-19 pandemic, despite the availability of technical support [27]. In the present study, the interview results reveal that the managers of the physiotherapy departments comprehended the need for telerehabilitation services during the pandemic, so some of their departments had started offering telerehabilitation services in order to prevent the spread of COVID-19, as well as to reduce waiting lists, facilitate patients’ access, and decrease patients’ transport time to attend clinics. Similarly, in a qualitative study conducted in Australia, the majority of healthcare providers said that telerehabilitation sessions would be a suitable option for some patients with a history of neurosurgery or orthopaedic issues, as the patients would not be required to travel to receive treatment [10].

### Comfort with technology

The results show that many of the PTs were happy using ICT for the purposes of patient care and education. They trusted telerehabilitation technology to enable them to provide physiotherapy services remotely. The findings also show that there was no association between comfort with technology and the PT’s age. However, the older PTs were more comfortable with using telerehabilitation systems. The interviews’ results support the overall trust in telerehabilitation technology by showing that PTs of different ages were willing to offer telerehabilitation services. With time and practice, they had become more motivated towards and interested in using telerehabilitation systems. This was supported by a previous study, which found that healthcare providers become more familiar with using technology and more comfortable with delivering general healthcare over time [12]. In contrast, the interview results from the present study reveal that elderly and illiterate patients face difficulties with telerehabilitation systems. A similar result was found in Nigeria, where PTs reported a low level of literacy among their patients in using telecommunication tools [14]. Thus, the authors recommended designing an effective means of educating potential patients, together with awareness campaigns [14]. In addition, they recommended that the hospital should encourage patients to take part in the tele-physiotherapy service [14].

### Willingness to use telerehabilitation

This study revealed that the majority of the PTs were willing to use telerehabilitation for practising physiotherapy at a distance and showed positive perceptions towards using video-calls with patients or with colleagues. The majority of the PTs agreed that telerehabilitation systems could be integrated with existing conventional systems; however, some of the PTs still preferred to practise physiotherapy via conventional in-person methods. This could be due to the nature of the physiotherapy profession, which requires a physical presence and hands-on interventions. This was also confirmed through the interviews, in which the managers stated that the PTs were happy and satisfied with telerehabilitation but that it is imperative that patients have their initial assessments in the clinic and then continue remotely as follow-up due to the difficulty in practising some assessments and treatments through telerehabilitation systems. This idea was consistent with previous studies, in which the healthcare providers preferred the initial appointment to be face to face, in order to establish patient–clinician rapport and practise objective hands-on techniques [10, 14, 16]. It is challenging to practise hands-on skills through telehealth systems; therefore, it is imperative to introduce efficient training on advanced technology tools, as suggested in previous studies [10, 11, 14].

### Barriers to the successful use of telerehabilitation

It is crucial to identify the barriers to the implementation of telerehabilitation systems to help eliminate them. In this study, a lack of technological readiness was the top barrier to telerehabilitation use, which included a lack of equipment and networks, a lack of user-friendly software, and a lack of connection between ICT experts and clinicians. Previous studies have reported that inadequate infrastructure (such as poor internet coverage, inadequate internet services, or network failure), the unavailability of telecommunication devices, and a lack of computer literacy are also barriers to the implementation of telerehabilitation [9, 10, 16, 17, 19]. The presence of specialists in health informatics could overcome these deficiencies. A lack of suitable training in the use of equipment to practise telerehabilitation was another barrier that was highlighted by most of the PTs, as technology barriers are considered a major issue in implementing telehealth systems [28]. In Saudi Arabia, PTs reported a lack of technical and staff skills as the main barriers to telerehabilitation [27]. The physiotherapy managers confirmed these barriers, as well as the disadvantages of telerehabilitation, which include a lack of hands-on skills, difficulty in practising real physiotherapy via video-calls, and the inability to create the same level of trust between specialists and patients. This could indicate that a lack of experience in using telerehabilitation made the specialists fear using it. Similarly, previous studies have shown that technically challenged staff are a major barrier to the adoption of telehealth [29–31]. A systematic review indicated that the strongest barriers are technology related but can be overcome through training [27]. Therefore, training programmes may help in overcoming technology-related barriers, and this concept was also emphasized by the managers regarding the need for awareness-raising initiatives to improve the telerehabilitation practices among PTs during and after the COVID-19 pandemic. Patient privacy and the confidentiality of their data were also significant concerns that the PTs considered as barriers to the use of telerehabilitation. This issue was reported in previous studies that found that patient safety and privacy are issues that could be compromised during the delivery of teleservices [10, 11, 13]. A couple of managers also highlighted staff privacy issues, where some female PTs refused to reveal themselves during video-calls in the presence of male caregivers. This could be resolved by promoting and designing policies and regulations to protect staff and ensure the safe practice of telerehabilitation, and this notion was stressed by the managers.

### Study strengths and limitations

This study was the first to be conducted with PTs from all specialties from governmental hospitals in Kuwait. This study had limitations: (1) the questionnaire return rate was low, which may have resulted in estimates that were biased by selective non-response; (2) the study did not include PTs working in private hospitals or clinics because the private sector has its own policies and regulations, which differ from those of the public sector. Thus, the findings cannot be generalized beyond the study sample; and (3) the study relied on self-reported data; thus, the PTs might have over-reported some information. However, the qualitative results support the overall findings in several ways.

## Conclusion

The impact of the COVID-19 pandemic has forced the physiotherapy profession to consider the risks associated with reducing healthcare and rehabilitation services, as well as consider alternative modes to deliver healthcare, such as telerehabilitation. Telehealth has been broadly used, and its impact on users and providers has been explored by many researchers. However, to date, no other studies have investigated the impact of such services among PTs in Kuwait during the COVID-19 pandemic. This study showed that most of the PTs were willing to use telerehabilitation, but they lacked support and proper guidance at work and needed continuous education programmes, regardless of their positive perceptions of and willingness to use telerehabilitation practices during and even after the COVID-19 pandemic. Accordingly, several recommendations are suggested.

### Study recommendations

This study has provided preliminary information regarding the use of telerehabilitation in physiotherapy departments. Healthcare decision-makers should consider the use of telerehabilitation and should design guidelines and policies to manage the telerehabilitation practices in Kuwait. Therefore, to facilitate the implementation of telerehabilitation in hospitals, it is recommended that support be provided from the technical side to address the technological requirements and from the organizational side to set up the required policies. Based on the findings of this study, suggested solutions are recommended for hospital managers, physiotherapy directors, and patients (see Additional file 2).

## Supplementary Information

**Additional file 1.** Questionnaire.

**Additional file 2.** Suggested solutions.

## Data Availability

The datasets used and/or analysed during the current study are available from the corresponding author on reasonable request.
