# Supplementary material for: Physiotherapists’ perceptions of and willingness to use telerehabilitation in Kuwait during the COVID-19 pandemic
Source: BMC Med Inform Decis Mak. 2021 Apr 8;21:122. doi: 10.1186/s12911-021-01478-x (PMC8028577; doi:10.1186/s12911-021-01478-x)
Supplement: Supplementary file 1 — Additional file 1. Questionnaire. [file 12911_2021_1478_MOESM1_ESM.pdf]

A questionnaire to Investigate Physiotherapists' Perceptions of and Willingness to Use Telerehabilitation in Kuwait During the COVID-19 Pandemic

Section 1: Socio-demographic data

1- **Age**:

- less than 35 years old
- 35 - 50
- 51 - 60
- Above 60

2- **Gender**:

- Female
- Male

3- **Nationality**

- Kuwaiti
- Non-Kuwaiti

4- Which hospital do you work?

- General hospital
- Rehabilitation hospital
- Specialist hospital

5- Years of experience:

- 10 years (PT practitioner / senior PT practitioner)
- 11 - 20 years (PT specialist / senior PT specialist)
- 21years and above (Superintendent PT)

Section 2: Technological background information

6- Do you use a computer at work?

☐1 Always ☐2 Often ☐3 Sometimes ☐4 Rarely ☐5 Never

7- Do you use internet at work?

☐1 Always ☐2 Often ☐3 Sometimes ☐4 Rarely ☐5 Never

8- Do you use e-mail at work?

☐1 Always ☐2 Often ☐3 Sometimes ☐4 Rarely ☐5 Never

9- Do you used any of telerehabilitation techniques in your work place?

☐1 Always ☐2 Often ☐3 Sometimes ☐4 Rarely ☐5 Never

Section 3: Perception about telerehabilitation system

10- Telerehabilitation system could be a solution for patients with physical problems during the pandemic.

☐1 Strongly disagree ☐2 Disagree ☐3 Agree ☐4 Strongly agree

11- Telerehabilitation is a viable approach for providing medical care services to patients.

☐1 Strongly disagree ☐2 Disagree ☐3 Agree ☐4 Strongly agree

12- ICT/ the internet has a potential role in healthcare.

☐1 Strongly disagree ☐2 Disagree ☐3 Agree ☐4 Strongly agree

13- Using of telerehabilitation system can save time, and money.

☐1 Strongly disagree ☐2 Disagree ☐3 Agree ☐4 Strongly agree

14- Telerehabilitation system can save efforts.

☐1 Strongly disagree ☐2 Disagree ☐3 Agree ☐4 Strongly agree

15- The application of ICT in health care is not available in our hospital.

☐1 Strongly disagree ☐2 Disagree ☐3 Agree ☐4 Strongly agree

Section 4: Comfort with technology

16- I can trust the technology to work.

☐1 Strongly disagree ☐2 Disagree ☐3 Agree ☐4 Strongly agree

17- I am happy in using ICT/Internet for the purpose of patient care and education.

☐1 Strongly disagree ☐2 Disagree ☐3 Agree ☐4 Strongly agree

18- There is a general comfort using ICT/Internet for storing, retrieving, and communicating of patient information with other health institutions.

☐1 Strongly disagree ☐2 Disagree ☐3 Agree ☐4 Strongly agree

19- Our culture and social norms do not prohibit the use of telerehabilitation systems.

☐1 Strongly disagree ☐2 Disagree ☐3 Agree ☐4 Strongly agree

Section 5: Willingness about telerehabilitation

20- I would be happy to use telerehabilitation to obtain consultations from other hospitals.

☐1 Strongly disagree ☐2 Disagree ☐3 Agree ☐4 Strongly agree

21- I would be happy to use telerehabilitation to watch live physiotherapy sessions as they take place

☐1 Strongly disagree ☐2 Disagree ☐3 Agree ☐4 Strongly agree

22- Telerehabilitation is appropriate in my hospital, based on current conditions

☐1 Strongly disagree ☐2 Disagree ☐3 Agree ☐4 Strongly agree

23- My colleagues would be willing to use telerehabilitation.

☐1 Strongly disagree ☐2 Disagree ☐3 Agree ☐4 Strongly agree

24- I would be happy to use telerehabilitation to deliver physiotherapy

☐1 Strongly disagree ☐2 Disagree ☐3 Agree ☐4 Strongly agree

25- Which of the following barriers to the use of telerehabilitation systems?

(Tick all that apply)

- o High cost of equipment
- o Negative attitudes of staff involved
- o Lack of suitable training to practice telerehabilitation
- o Perceived increase in workload
- o Patient privacy and confidentiality of their data
- o Lack of user-friendly software
- o Lack of perceived clinical usefulness
- o Lack of connection between ICT experts and clinicians
- o Others, please specify……………………………………….

Thank you for your participating
