# Supplementary material for: Physiotherapists’ perceptions of and willingness to use telerehabilitation in Kuwait during the COVID-19 pandemic
Source: BMC Med Inform Decis Mak. 2021 Apr 8;21:122. doi: 10.1186/s12911-021-01478-x (PMC8028577; doi:10.1186/s12911-021-01478-x)
Supplement: Supplementary file 2 — Additional file 2. Suggested solutions. [file 12911_2021_1478_MOESM2_ESM.pdf]

Physiotherapists' Perceptions of and Willingness to Use Telerehabilitation in Kuwait during the COVID-19 Pandemic

Sarah Ibraheem Albahrouh[1] and Ali Jasem Buabbas[2]*

[1] Department of Physical Therapy, Faculty of Allied Health Sciences, Kuwait University

[2] Department of Community Medicine, Faculty of Medicine, Kuwait University

***Corresponding Author**

Ali Jasem Buabbas,

Correspondence to Email: ali.buabbas@hsc.edu.kw

Additional file 1

Title: Suggested solutions

Barriers	Solutions
Increased staff work overload	• Two timetables should be developed to arrange PTs work: One for direct patients' care of physical therapy, and one for practicing telerehabilitation only
Refuse to use telerehabilitation (staff resistance)<br>Lack of technology trust<br>Lack of knowledge about role of technology in rehabilitation care	• Awareness is very important for both, patients and clinical staff, where patient's consent is obligatory to be taken when using the technology in practice. Therefore, awareness sessions to demonstrate the role of technology in health care, specifically about telehealth use.
Lack of perceived clinical usefulness	• It is important to learn from successful applications of telerehabilitation in clinical practice and to trial systems for specific cases to ensure the effectiveness of applications.<br>• Telerehabilitation systems should be evaluated in terms of their efficiency and effectiveness in practice.
Lack of connection between ICT experts and clinicians	• It is recommended to create and maintain connections between IT experts and PTs specialist. Specialists in health informatics should be involved to manage this process.
Patient privacy and confidentiality of their data	• Guidelines should be developed to: (1) suggest high quality security applications that can be uploaded into smartphone devices, in order to be make them eligible for hospital-related work; (2) suggest a number of trusted smartphone applications that can be used for medical practice (3) maintain the legal right for patients and clinicians alike.
Lack of training courses on using telerehabilitation system	• Continuous training sessions should be provided to ensure the optimum use of

	telerehabilitation systems and to avoid obstacles such as staff resistance.
Uunavailability of adequate equipment, poor network coverage, and a lack of hospital of IT support	• Information technology support should be offered to train the medical staff the different functionalities of the smart technology for clinical use, and technical requirements should be offered in place, such as the Wi-Fi connection, battery life, etc.
Lack of guidelines, policies, and protocols for telehealth use	• Hospital management should have a crucial role in managing the practice of telehealth that supported by mobile technology devices, considering developing guidelines to protect both patients and staff from legal allegations and to ensure safe and effective practice of telerehabilitation.<br>• Importantly, the quick start guide for telehealth during a health crisis developed by the American Telemedicine Association should be adopted [32].<br>• Integration to electronic patient records (EPR): it's very important to give the access for medical staff to the EPR via mobile technology, to have a complete picture about the patient case in order to make an accurate clinical decision.
Elderly and poor educated patients are not willing to receive tele rehabilitation care	• To promote the use of telehealth among patients, and particularly this category, awareness is important about the role of technology in health care via bulletin boards, brochures, and roll-up banners in different health care settings.<br>• Also, patients would be advised to bring one of their relatives during the virtual session to help them out with technology use.<br>• A quick workshop on using mobile technology in health care should be offered by the specialists to their patients to improve their digital literacy.
